# Associations between age and gray matter volume in anatomical brain networks in middle-aged to older adults

**DOI:** 10.1111/acel.12271

**Published:** 2014-09-25

**Authors:** Anne Hafkemeijer, Irmhild Altmann-Schneider, Anton J M de Craen, P Eline Slagboom, Jeroen van der Grond, Serge A R B Rombouts

**Affiliations:** 1 Institute of Psychology, Leiden UniversityLeiden, The Netherlands; 2 Department of Radiology, Leiden University Medical CenterLeiden, The Netherlands; 3 Leiden Institute for Brain and Cognition, Leiden UniversityLeiden, The Netherlands; 4 Department of Molecular Epidemiology, Netherlands Consortium for Healthy Ageing, Leiden University Medical CenterLeiden, The Netherlands; 5 Department of Gerontology and Geriatrics, Leiden University Medical CenterLeiden, The Netherlands; 6 Department of Molecular Epidemiology, Leiden University Medical CenterLeiden, The Netherlands

**Keywords:** aging, atrophy, brain, gray matter, magnetic resonance imaging, structural covariance networks

## Abstract

Aging is associated with cognitive decline, diminished brain function, regional brain atrophy, and disrupted structural and functional brain connectivity. Understanding brain networks in aging is essential, as brain function depends on large-scale distributed networks. Little is known of structural covariance networks to study inter-regional gray matter anatomical associations in aging. Here, we investigate anatomical brain networks based on structural covariance of gray matter volume among 370 middle-aged to older adults of 45–85 years. For each of 370 subjects, we acquired a T1-weighted anatomical MRI scan. After segmentation of structural MRI scans, nine anatomical networks were defined based on structural covariance of gray matter volume among subjects. We analyzed associations between age and gray matter volume in anatomical networks using linear regression analyses. Age was negatively associated with gray matter volume in four anatomical networks (*P* < 0.001, corrected): a subcortical network, sensorimotor network, posterior cingulate network, and an anterior cingulate network. Age was not significantly associated with gray matter volume in five networks: temporal network, auditory network, and three cerebellar networks. These results were independent of gender and white matter hyperintensities. Gray matter volume decreases with age in networks containing subcortical structures, sensorimotor structures, posterior, and anterior cingulate cortices. Gray matter volume in temporal, auditory, and cerebellar networks remains relatively unaffected with advancing age.

## Introduction

It is well recognized that the process of aging is associated with cognitive decline and diminished brain function (Grady, 2012). In addition, numerous neuroimaging studies have unequivocally shown that aging is associated with loss of brain tissue, in which process especially the gray matter seems affected. Volumetric and morphometric neuroimaging studies have demonstrated a consistent age-dependent decrease in regional gray matter volume, mainly expressed in the temporal lobe and hippocampus, the cingulate cortex, and prefrontal regions (Good *et al*., 2001; Jernigan *et al*., 2001; Resnick *et al*., 2003; Raz *et al*., 2005).

There is increasing evidence that, in addition to brain atrophy, aging and loss of cognitive function at high ages are associated with disrupted structural and functional brain connectivity. It has been shown that functional connectivity decreases with age, especially connectivity in the default mode network between the medial prefrontal cortex, anterior and posterior cingulate cortex, precuneus, parietal cortex, and hippocampus (Damoiseaux *et al*., 2008; Hafkemeijer *et al*., 2012; Ferreira & Busatto, 2013). Furthermore, aging is associated with disrupted white matter anatomical connections, specifically in the frontal white matter, anterior cingulum, and the genu of the corpus callosum (Salat *et al*., 2005; Madden *et al*., 2012).

In addition to functional brain networks and white matter anatomical connectivity, population (intersubject) covariance of gray matter volume can be used to study inter-regional anatomical associations (Alexander-Bloch *et al*., 2013). The integrity of these gray matter structural covariance networks changes throughout lifespan (Wu *et al*., 2012, 2013). Here, we will investigate the integrity of gray matter anatomical networks in the aging brain. In this respect, mainly the structural covariance of the default mode network has been studied, showing a breakdown with increasing age (Spreng & Turner, 2013). While most studies focused on the default mode network, there is evidence for age-dependent decreases in other anatomical networks (Montembeault *et al*., 2012; Segall *et al*., 2012; Li *et al*., 2013).

Currently, anatomical networks are mostly studied using a model-driven seed-based approach with *a priori* hypotheses of manually selected regions of interest and their connected networks (Montembeault *et al*., 2012; Zielinski *et al*., 2012; Li *et al*., 2013; Soriano-Mas *et al*., 2013). The manual selection of regions of interest might introduce a selection bias (Damoiseaux & Greicius, 2009). To avoid this, we will use a model-free method to investigate whole-brain anatomical networks in an unrestricted exploratory way. This method has proven to be a powerful tool to characterize structural networks in schizophrenia (Xu *et al*., 2009). Here, we will apply this method to study gray matter anatomical networks in middle-aged to older adults.

In this study, we explored anatomical networks in a large group of middle-aged to older adults (45–85 years, *n* = 370). Our aim was to investigate whole-brain anatomical networks to explore which networks are associated with the process of healthy aging and which networks do not show an age association.

## Results

### Demographic characteristics

We analyzed structural MRI scans of in total 370 middle-aged to older participants aged between 45.5 and 84.3 years (mean age 65.7 ± 6.7 years). The cohort was nearly balanced on gender (192 women, 51.9%), with similar age distributions across genders. The study population has been described in more detail elsewhere (Altmann-Schneider *et al*., 2013).

The total study population was divided into four age subgroups: (i) 45–55 years, mean age = 51.4 ± 2.3 years, *n* = 26, 18 women; (ii) 55–65 years, mean age = 61.5 ± 2.4 years, *n* = 145, 83 women; (iii) 65–75 years, mean age = 69.3 ± 2.7 years, *n* = 171, 83 women; and (iv) 75-85 years, mean age = 78.0 ± 2.6 years, *n* = 28, 8 women. White matter hyperintensities (WMHs) were defined as areas within the cerebral white matter with increased signal intensity. Mean volume of WMHs was 1.93 mL for the total study population [0.81 mL (45–55 years), 1.25 mL (55–65 years), 1.93 mL (65–75 years), 5.99 mL (75–85 years)].

### Gray matter anatomical brain networks

**Figure 1:**
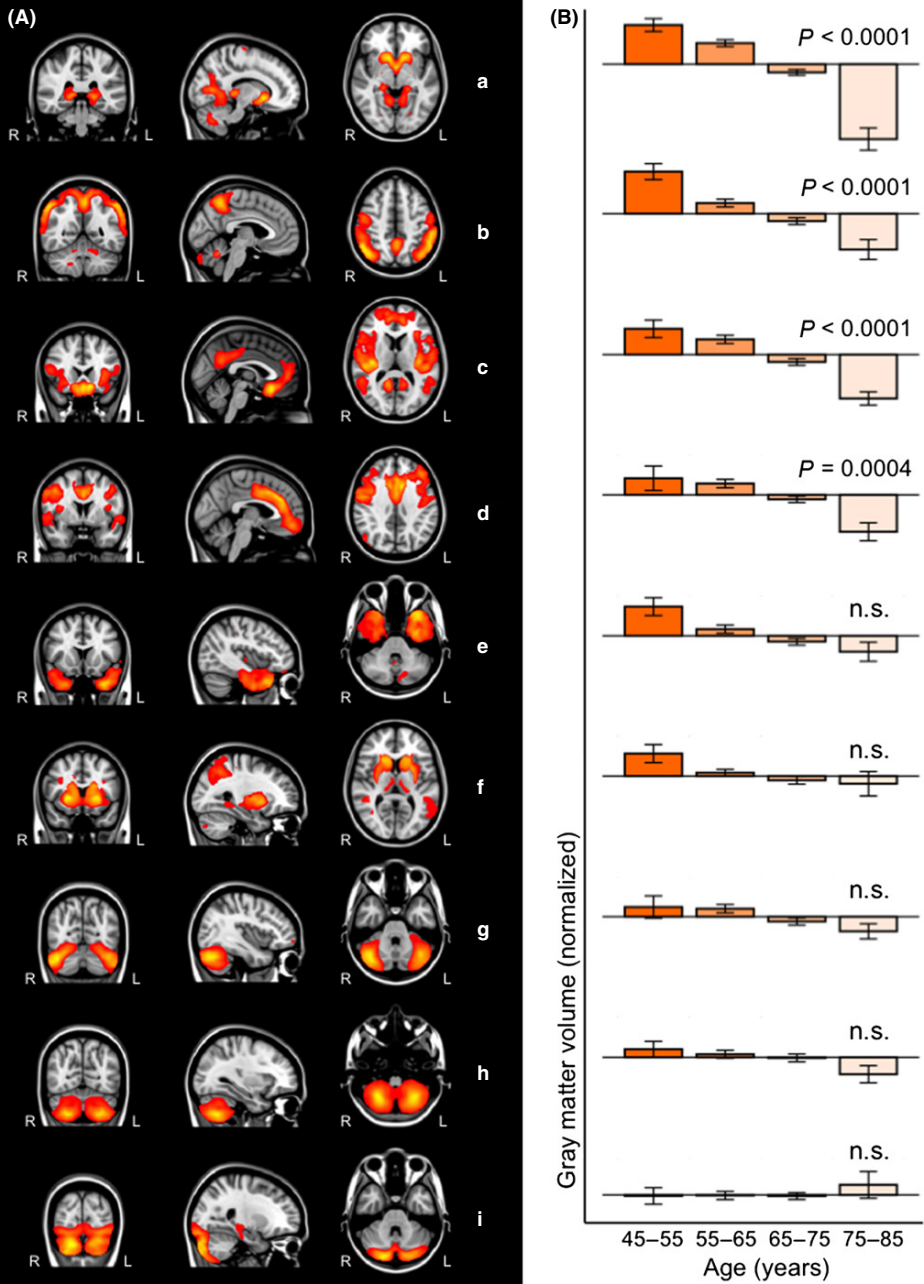
Gray matter structural networks and associations with age. (A) Overview of the nine anatomical networks based on the covariation of gray matter volumes among middle-aged to older adults. Networks are overlaid on the most informative coronal, sagittal, and transverse slices of the MNI-152 standard anatomical image. (B) The association between age and gray matter volume in the anatomical networks is illustrated by bar graphs. Error bars indicate the standard error of the mean. Age was negatively associated with gray matter volume in network a–d and was not significantly associated with gray matter volume in network e–i.

After segmentation of structural MRI scans, gray matter images were used to define nine anatomical brain networks based on the covariation of gray matter volumes among the middle-aged to older adults (Fig.1A). Brain structures of the networks were identified using the Harvard-Oxford atlas integrated in Functional Magnetic Resonance Imaging of the Brain Software Library (FSL) (Table1).

**Table 1:** Brain clusters of anatomical brain networks

	Brain cluster†	Cluster volume	MNI coordinates		
		(cm^3^)	x	y	z
Network a	Thalamus	17.90	−2	−2	−8
	Cluster also contains nucleus accumbens, caudate nucleus, hippocampus, lingual gyrus, and cerebellum				
	(Postcentral gyrus)	1.03	52	−8	32
	(Precentral gyrus)	0.89	−20	−18	70
	(Heschl's gyrus)	0.41	−50	−26	10
Network b	Lateral occipital cortex	36.76	50	−62	44
	Cluster also contains precuneus and supramarginal gyrus				
	Cerebellum	3.17	−18	−72	−34
Network c	Posterior cingulate cortex	56.75	−8	22	−16
	Cluster also contains paracingulate gyrus, subcallosal cortex, operculum cortex, and precuneus				
	Middle temporal gyrus	6.32	56	−48	8
	(Occipital fusiform gyrus)	0.42	26	−74	−14
	Lateral occipital cortex	0.28	−40	−72	26
Network d	Anterior cingulate cortex	36.81	−2	32	28
	Cluster also contains middle frontal gyrus, precentral gyrus, and frontal medial cortex				
	(Cerebellum)	3.06	−20	−80	−44
	(Lateral occipital cortex)	2.47	50	−74	26
	(Temporal pole)	1.56	−58	6	−2
	(Cuneus)	0.84	12	−68	24
	(Precuneus)	0.68	−14	−62	22
Network e	Temporal pole	29.04	−32	22	−38
	Cluster also contains temporal fusiform cortex				
	(Cerebellum)	2.59	−12	−74	−30
	(Anterior cingulate cortex)	1.57	10	−12	44
Network f	Putamen	18.74	24	14	0
	Cluster also contains caudate nucleus (and insular cortex)				
	Superior parietal lobule	10.40	34	−48	38
	Cluster also contains lateral occipital cortex (and precuneus)				
	(Cerebellum)	5.37	−6	−66	−16
	Angular gyrus	5.35	−44	−58	20
Network g	Cerebellum	24.35	42	−68	−32
	(Frontal pole)	0.41	52	34	−6
Network h	Cerebellum	30.43	26	−64	−52
	(Middle frontal gyrus)	0.57	−50	28	24
	(Precuneus)	0.90	20	−58	20
Network i	Cerebellum	25.18	18	−86	−36
	Hippocampus	0.49	24	−24	−8
	(Postcentral gyrus)	0.49	−40	−30	40
	(Frontal pole)	0.28	8	64	12

MNI, Montreal Neurological Institute 152 standard space image.

† Each gray matter anatomical network is divided into brain clusters using the cluster tool integrated in FSL. Cluster size and MNI x-, y-, and z-coordinates of each cluster are given. Brain structures are anatomically identified using the Harvard-Oxford atlas integrated in FSL. Fig.1 shows the most informative coronal, sagittal, and transverse slices. Structures in parentheses in the table are not visible in Fig.1.

### Aging

To analyze the possible association between age and gray matter volume in anatomical networks, we used a linear regression analysis based on four age subgroups (45–55, 55–65, 65–75, and 75–85 years). To statistically account for the possible influences of gender, family characteristics (i.e., offspring of long-lived parents or nonoffspring), and volumes of WMHs, these factors were used as independent factors in the linear regression model. The age association of gray matter volume in anatomical networks is illustrated in Fig.1B. (This figure shows the networks in order of age association, with the first network showing the strongest association with age.)

Age showed a negative association with gray matter volume in four networks (Fig.1A–D). These networks included 1) thalamus, nucleus accumbens, caudate nucleus, hippocampus, and lingual gyrus (network a in Fig.1 and Table1, *P* < 0.0001, *R*^2^ = 0.291, Beta = −0.510), 2) lateral occipital cortex and precuneus (network b in Fig.1 and Table1, *P* < 0.0001, *R*^2^ = 0.257, Beta = −0.255), 3) posterior cingulate cortex, paracingulate gyrus, subcallosal cortex, and operculum cortex (network c in Fig.1 and Table1, *P* < 0.0001, *R*^2^ = 0.158, Beta = −0.347), and 4) anterior cingulate cortex, middle frontal gyrus, and frontal medial cortex (network d in Fig.1 and Table1, *P* = 0.0004, *R*^2^ = 0.150, Beta = −0.186).

Age was not significantly associated with gray matter volume in five networks (Fig.1E–I). These networks included 1) temporal pole and temporal fusiform cortex (network e in Fig.1 and Table1, *P* = 0.0038, *R*^2^ = 0.085, Beta = −0.157), 2) putamen, caudate nucleus, and superior parietal lobule (network f in Fig.1 and Table1, *P* = 0.0137, *R*^2^ =0.083, Beta = −0.134), and 3–5) three cerebellar networks (network g in Fig.1 and Table1, *P* = 0.1709, *R*^2^ = 0.078, Beta = −0.074; network h in Fig.1 and Table1, *P* = 0.3496, *R*^2^ = 0.020, Beta = −0.052; network i in Fig.1 and Table1, *P* = 0.1233, *R*^2^ = 0.030, Beta = 0.086).

### Longevity

All subjects were included from the Leiden Longevity Study, which was set up to identify genetic and phenotypic markers related to longevity (Altmann-Schneider *et al*., 2013). The study cohort consists of offspring of long-lived siblings and their partners (194 offspring and 176 partners). The offspring was characterized by having long-lived parents (with male parents aged ≥ 89 years and female parents aged ≥ 91 years). No significant differences in association between age and the gray matter volume in the anatomical networks were found between offspring of long-lived parents and nonoffspring participants.

## Discussion

We identified anatomical brain networks based on structural covariance of gray matter volume in a large sample of healthy participants aged between 45 and 85 years. Our aim was to investigate whole-brain anatomical networks to explore which networks are associated with the process of healthy aging and which networks do not show an age association. In summary, by doing a cross-sectional analysis, we found gray matter volume decreases with age in four networks containing predominantly subcortical structures, lateral occipital, posterior, and anterior cingulate cortices. The gray matter in five networks containing the temporal pole, putamen, and cerebellum remained relatively unaffected with advancing age.

The greatest associations with age were found in an anatomical network containing among other structures the thalamus, nucleus accumbens, caudate nucleus, and hippocampus (network a). It is well recognized that subcortical structures are vulnerable to atrophy with advancing age (Jernigan *et al*., 2001; Raz *et al*., 2005). Additionally, network studies have shown age-dependent relationships between these structures (Brickman *et al*., 2007; Bergfield *et al*., 2010; Soriano-Mas *et al*., 2013).

We found associations between age and the gray matter volume in the lateral occipital network (network b). Evidence for age associations of gray matter volume in this anatomical network is supported by others (Montembeault *et al*., 2012; Li *et al*., 2013). Montembeault et al. found reduced structural associations between occipital regions and the temporal pole (Montembeault *et al*., 2012). That finding is consistent with disrupted white matter anatomical connections between occipital and temporal areas in the elderly (Kantarci *et al*., 2011). It has been suggested that these age-related reduced structural associations may explain the decline in language-related semantics in the elderly (Montembeault *et al*., 2012).

In general, gray matter anatomical networks and resting state functional connectivity networks spatially overlap (Seeley *et al*., 2009; Segall *et al*., 2012) and reflect regions that co-degenerate in several neurodegenerative syndromes (Seeley *et al*., 2009). Our study also shows spatial overlap between the structures of anatomical networks and resting state functional connectivity networks found in other studies (Beckmann *et al*., 2005; Damoiseaux *et al*., 2006; Laird *et al*., 2011). Visual inspection shows spatial overlap between network a and the medial visual cortical functional connectivity network, network b and sensorimotor functional connectivity network, network c and default mode network, network d and executive control network, network e and medial temporal functional connectivity network, and network f and the auditory functional connectivity network. The cerebellum (network g, h, and i) is less frequently studied with functional connectivity.

The association with age we found in the posterior cingulate anatomical network (network c) is consistent with a recent study that showed that this anatomical network changes with age in healthy and pathological aging (Spreng & Turner, 2013). Visual inspection of our data showed spatial overlap between the structures of this anatomical network and the default mode functional connectivity network found in other studies (Beckmann *et al*., 2005; Damoiseaux *et al*., 2006; Laird *et al*., 2011). The default mode network is affected by age-related atrophy (Buckner *et al*., 2008) and age-related decreases in functional connectivity (Damoiseaux *et al*., 2008; Hafkemeijer *et al*., 2012; Ferreira & Busatto, 2013).

The anatomical network containing predominantly the anterior cingulate cortex (network d) shows spatial overlap with a functional connectivity network associated with executive control functions (Beckmann *et al*., 2005; Damoiseaux *et al*., 2006; Laird *et al*., 2011). The associations between age and gray matter volume in this network is supported by other anatomical network studies (Bergfield *et al*., 2010; Montembeault *et al*., 2012). It has been suggested that the age-dependent breakdown of this network may explain the difficulties in cognitively demanding tasks generally observed in elderly (Montembeault *et al*., 2012).

Prior studies mostly focused on age-related differences in the aging brain. Relatively few studies have sought to identify anatomical networks that were not associated with age. Functional connectivity in somatosensory and cerebellar networks does not show an association with advancing age (Tomasi & Volkow, 2012). Here, we showed that gray matter volume in five anatomical networks, predominantly containing the temporal pole (network e), putamen (network f), and cerebellum (networks g, h, and i), was not associated with age. The lack of age associations in these five networks is in line with an anatomical network study in healthy elderly (Bergfield *et al*., 2010). Others have shown that the temporal areas, putamen, and cerebellum are less susceptible to age-related differences in both gray matter volume and metabolism (Kalpouzos *et al*., 2009). However, age-related differences in the temporal anatomical network are frequently reported (Alexander *et al*., 2006; Brickman *et al*., 2007; Montembeault *et al*., 2012), which makes preservation of this network more unlikely. In this study, we found a nonsignificant trend toward age-related gray matter volume loss in the temporal network (network e). Further research is highly recommended to investigate the association between age and the gray matter volume in the temporal anatomical network.

Here, we studied whole-brain anatomical networks. The method used in this study examines the inter-regional anatomical relationship among spatially distributed brain structures as networks of connected regions. This approach showed associations between age and gray matter networks containing brain areas that were found earlier in several other studies exploring regional (non-network) gray matter differences. This suggests a high sensitivity of the network approach used in our study. However, this interpretation should be taken with caution, given the lack of direct comparisons between non-network and network studies and given the differences in statistical correction for multiple comparisons (i.e., network studies should correct for multiple networks, whereas regional non-network studies are forced to use a more stringent voxelwise correction for multiple comparisons).

In this study, we used the ICA method to determine anatomical networks based on the covariation of gray matter volumes among all 370 middle-aged to older adults. The age associations reported in our study might be influenced by the fact that the anatomical networks are based on the total study population of middle-aged to older adults. Although much can be learned from the age associations found in our study, a limitation of this cross-sectional study is that the participants were not followed over time. A longitudinal design is required to study changes in individual brain structure as aging occurs.

Another limitation is that the number of components to estimate (i.e., anatomical networks) is arbitrarily chosen. The topic of choosing the number of components and the effect of the dimensionality on the statistical results is currently an active area of research. There is no consensus on the optimal number of components (Cole *et al*., 2010), which may vary depending on the data and the research question. In the current study, we decided to use a dimensionality within the range of the most often applied dimensionality in studies of brain networks, that is use eight to ten components (Beckmann *et al*., 2005; Damoiseaux *et al*., 2006; Smith *et al*., 2009; Segall *et al*., 2012; Zielinski *et al*., 2012). However, it is important to note that varying the dimensionality may affect the sensitivity to detect regional effects and may impact the findings of this study.

Overall, we showed that regionally separate gray matter regions are organized in anatomical networks. We gave an overview of associations between age and the gray matter volume in these networks. Elderly show a decline in gray matter volume in networks containing subcortical structures, lateral occipital, posterior, and anterior cingulate cortices. Anatomical networks containing the temporal pole, putamen, and cerebellum remain relatively unaffected with advancing age. The current work supports the application of gray matter structural network analysis to evaluate inter-regional anatomical relationships among spatially distributed brain structures in the aging brain. Additionally, this approach may also be useful in distinguishing the effects of age-related neurodegenerative disease from healthy aging.

## Experimental procedures

### Participants

In total, 370 subjects aged between 45 and 85 years were included from the Leiden Longevity Study, which was set up to identify genetic and phenotypic markers related to longevity (Altmann-Schneider *et al*., 2013). For the current study, the offspring of long-lived siblings and their partners were included (194 offspring and 176 partners). The offspring is characterized by having long-lived parents (with male parents aged ≥ 89 years and female parents aged ≥ 91 years). The cohort is nearly balanced on gender (192 females, 51.9%), with similar age distributions across genders.

All subjects underwent an extensive medical screening. Cognitive functioning was assessed by a neuropsychological protocol. The participants did not demonstrate any abnormalities on neuropsychological elevation (Mini-Mental State Examination score > 28, Geriatric Depression Scale-15 score < 6) and did not have a history of psychiatric or neurodegenerative disorders. In accordance with the Declaration of Helsinki, written informed consent from all participants was obtained. The Medical Ethical Committee of the Leiden University Medical Center approved the study.

### Data acquisition

All participants underwent an MRI of the brain in the Leiden University Medical Center. Imaging was performed on a Philips 3 Tesla Achieva MRI scanner using a standard whole-head coil for radiofrequency transmission (Philips Medical Systems, Best, the Netherlands).

Three-dimensional T1-weighted anatomical images were acquired with the following parameters: TR = 9.7 ms, TE = 4.6 ms, flip angle = 8°, FOV = 224 × 177 × 168 mm, resulting in a nominal voxel size of 1.17 × 1.17 × 1.40 mm, covering the entire brain with no gap between slices. To determine WMHs, we acquired fluid-attenuated inversion recovery (FLAIR) images (TR = 11 000 ms, TE = 125 ms, flip angle = 90°, FOV = 220 × 176 × 137 mm, matrix size 320 × 240, 25 transverse slices to cover the entire brain with a slice thickness of 5 mm with no gap between slices).

### Gray matter anatomical brain networks

Before analysis, all MRI scans were submitted to a visual quality control check to ensure that no gross artifacts were present in the data. Data analysis was performed with FSL (FSL 4.1.9, Oxford, United Kingdom, www.fmrib.ox.ac.uk/fsl.

First, nonbrain tissue (e.g., scalp) was removed from T1-weighted images using a semi-automated brain extraction tool as implemented in FSL (Smith, 2002). Next, tissue-type segmentation was performed using voxel-based morphometric analysis (Ashburner & Friston, 2000). We performed a control check after each processing step to ensure appropriate brain extraction and tissue-type segmentation. To correct for the partial volume effect (i.e., voxels ‘containing’ more than one tissue type), the tissue-type segmentation was carried out with partial volume estimation. For each partial volume voxel, the proportion of each tissue type was estimated, that is, a partial volume vector was formed, with each element being a ‘fraction’ of a specific tissue type and having a sum of one (Zhang *et al*., 2001). The segmented images have values that indicate the probability of a given tissue type (i.e., they are not binary).

The resulting gray matter partial volume images were aligned to the gray matter MNI 152 standard space image (Montreal Neurological Institute, Montreal, QC, Canada) (Jenkinson *et al*., 2002), followed by nonlinear registration (Andersson *et al*., 2007). The resulting images were averaged to create a study-specific gray matter template, to which the native gray matter segmented images were nonlinearly re-registered (Ashburner & Friston, 2000; Good *et al*., 2001). As a result of nonlinear spatial registration, the volume of some brain structures may grow, whereas others may shrink. To correct for these enlargements and contractions, a further processing step (modulation) is recommended (Ashburner & Friston, 2000; Good *et al*., 2001). In this additional step, each voxel of each registered gray matter image was divided by the Jacobian of the warp field, which defines the direction (larger or smaller) and the amount of modulation. The modulated segmented images were finally spatially smoothed with an isotropic Gaussian kernel with a sigma of 3 mm.

The modulated gray matter images in MNI space of all 370 subjects were used as a four-dimensional data set on which an independent component analysis (ICA) was applied using multivariate exploratory linear optimized decomposition into independent components (Beckmann *et al*., 2005). ICA is a statistical technique that decomposes a set of signals into spatial component maps of maximal statistical independence (Beckmann & Smith, 2004). When applied on gray matter images of different subjects, this method defines fully automatically spatial components based on the covariation of gray matter volumes among subjects (i.e., structural covariance networks), without *a priori* selected regions of interest. A limitation of this technique is that the number of components to estimate is arbitrarily chosen and that there is no consensus on how to choose the optimal number of components (Cole *et al*., 2010). There even exists no single ‘best’ dimensionality. Structural covariance and resting state functional networks are in general investigated using eight to ten components (Beckmann *et al*., 2005; Damoiseaux *et al*., 2006; Smith *et al*., 2009; Segall *et al*., 2012; Zielinski *et al*., 2012). Therefore, in this study, the ICA output was restricted to nine components.

A mixture model was used to assign significance to individual voxels within a spatial map, using a standard threshold level of 0.5 (Beckmann & Smith, 2004). This indicates that a voxel ‘survives’ thresholding as soon as the probability of being in the ‘nonbackground’ class exceeds the probability of being in the ‘background’ noise class. A threshold of 0.5 indicates that an equal loss is placed on false positives and false negatives. Anatomical locations were determined using the Harvard-Oxford atlas integrated in FSL.

### White matter hyperintensities

We statistically accounted for the possible influence of WMHs, as the prevalence of WMHs increases with age (Galluzzi *et al*., 2008). Furthermore, the degree of white matter damage is associated with a decrease in gray matter volume in healthy elderly (Wen *et al*., 2006). The anatomical locations of the networks were identified based on the covariance of gray matter; the presence of WMHs does not affect the identification of the networks. However, WMHs could be associated with the amount of gray matter within each individual network. Therefore, we added the volumes of WMHs as independent factor to the linear regression model (see section ‘Statistical analysis’). WMHs were defined as areas within the cerebral white matter with increased signal intensity on the FLAIR images. Volumes of WMHs were automatically determined using a previously validated method (King *et al*., 2013). In short, after tissue segmentation of the T1-weighted images, white matter masks generated by FSL were spatially transformed to the FLAIR images using FMRIB's Linear Image Registration Tool (Jenkinson *et al*., 2002). WMHs in the mask were automatically identified using a threshold of three standard deviations above the mean FLAIR signal intensity (King *et al*., 2013).

### Statistical analysis

To analyze the possible association between age and gray matter volume in anatomical networks, we used a linear regression analysis (IBM SPSS Statistics Version 20, IBM Corp., Somers, NY, USA). We divided the total age span four age subgroups (45–55, 55–65, 65–75, and 75–85 years) and used these four age subgroups as a categorical variable in the linear regression model. To statistically account for the possible influences of gender, family characteristics (i.e., offspring of long-lived parents or nonoffspring), and volumes of WMHs, these factors were used as independent factors in the model. The statistical threshold was corrected for multiple comparisons using the Bonferroni correction based on 2 × 9 = 18 comparisons (two-tailed, nine networks) yielding a corrected *P* value threshold of 0.05/18 = 0.003.

## Author contributions

A. Hafkemeijer, J. van der Grond, and S. Rombouts made substantial contributions to conception and design, analysis, and interpretation of the data. I. Altmann-Schneider, A. de Craen, P. Slagboom, and J. van der Grond made substantial contributions to acquisition of the data and interpretation of the data. All authors contributed to the drafting of the article and critically revised it.

## Conflict of interest

The authors report no conflict of interest. The Medical Ethical Committee of the Leiden University Medical Center approved the study, and written informed consent was obtained from all participants according to the Declaration of Helsinki.

## Funding

This work was supported by funding from the Netherlands Initiative Brain and Cognition (NIHC), a part of the Netherlands Organization for Scientific Research (NWO) (Grant Numbers 05613010, 91786368); the Innovation-Oriented Research Program on Genomics (Grant Number SenterNovem IGE05007); and the Netherlands Consortium for Healthy Ageing (Grant Number 050060810).

## Abbreviations

FLAIR: fluid-attenuated inversion recovery
FSL: functional magnetic resonance imaging of the brain software library
ICA: independent component analysis
MNI: Montreal Neurological Institute
WMHs: white matter hyperintensities
